# Genome editing iPSC to purposing enhancement of induce CD8 killer T cell function for regenerative immunotherapy

**DOI:** 10.1186/s41232-024-00328-3

**Published:** 2024-04-18

**Authors:** Sota Kurihara, Akihiro Ishikawa, Shin Kaneko

**Affiliations:** Shin Kaneko Laboratory, Department of Cell Growth and Differentiation, Center for iPS Cell Research and Application (CiRA), Kyoto University, Kyoto, Japan

**Keywords:** CD8 + T cell, Chimeric antigen receptor, Genome editing, Immunotherapy, iPSC

## Abstract

In recent years, immunotherapy has become a standard cancer therapy, joining surgery, chemotherapy, and radiation therapy. This therapeutic approach involves the use of patient**-**derived antigen**-**specific T cells or genetically modified T cells engineered with chimeric antigen receptors (CAR) or T cell receptors (TCR) that specifically target cancer antigens. However, T cells require ex vivo stimulation for proliferation when used in therapy, and the resulting “exhaustion,” which is characterized by a diminished proliferation capacity and anti**-**tumor activity, poses a significant challenge. As a solution, we reported “rejuvenated” CD8 + T cells that possess high proliferation capacity from induced pluripotent stem cells (iPSCs) in 2013. This review discusses the status and future developments in immunotherapy using iPSC**-**derived T cells, drawing insights from our research to overcome the exhaustion associated with antigen**-**specific T cell therapy.

## Utility and challenges of antigen-specific CD8 + T cells

The immune system, a vital component of the body’s defense mechanisms, functions through coordinated activities of diverse immune cells to eliminate foreign entities. T cells play a central role in acquired immunity and are categorized into three subsets: helper T cells (CD4 + T cells), cytotoxic T cells (CD8 + T cells), and regulatory T cells (CD4 + CD25 + T cells, or Treg cells) [1]. Antigen recognition by T cells is mediated by the T cell receptor (TCR) expressed on the cell surface; this TCR specifically binds to human leukocyte antigen (HLA) molecules. T cells undergo activation upon stimulation by antigen-presenting cells, such as dendritic cells. This activation is crucial for anti**-**tumor immune responses, with the CD4 + and CD8 + T cell subsets being particularly significant. CD4 + T cells serve as command centers in anti**-**tumor immune responses, orchestrating functions such as enhancing phagocytosis, promoting antibody production, and activating natural killer (NK) cells and CD8 + T cells. On the other hand, activated CD8 + T cells form immunological synapses with cancer cells, releasing cytotoxic granules, such as granzyme B, which directly attack cancer cells. In the realm of antitumor immune responses, the impact of CD8 + T cell**-**mediated attacks is substantial, and these cells are a major effector cell population. Notably, a subset of activated CD8 + T cells differentiate into memory T cells with heightened production capabilities of inflammatory cytokines, such as interferon**-**γ, tumor necrosis factor, and interleukin**-**2 (IL**-**2), and the ability to proliferate upon antigen recognition, resulting in a lasting presence in the body and rapid immune responses upon subsequent antigen exposures [2, 3]. However, T cells turn to the exhaustion status, in which their cytotoxic activity and inflammatory cytokine production are diminished when stimulated chronically [4–6]. Exhausted T cells express immune checkpoint molecules, such as programmed death receptor**-**1, lymphocyte**-**activation gene 3, and T cell immunoglobulin mucin**-**3, on their cell surfaces, resulting in suppressed activity [7]. Exhaustion not only compromises the ability of T cells to proliferate in response to antigens, but it also contributes to the depletion of antigen**-**specific T cells in the body, significantly impacting disease control [8, 9].

To address the qualitative and quantitative loss of antigen**-**specific T cells, research has explored the ex vivo amplification of antigen**-**specific T cells for infusion as well as the genetic introduction of antigen-specific TCRs or chimeric antigen receptors (CARs) to T cells for adoptive cell therapy. In 2011, Rosenberg et al. achieved a 22% complete response rate in melanoma patients by infusing autologous tumor**-**infiltrating lymphocytes (TILs) amplified in vitro [10]. CAR**-**T cell therapy targeting CD19 demonstrated strong antitumor efficacy in refractory and relapsed acute lymphoblastic leukemia (ALL) [11]. The success in achieving complete responses is attributed, in part, to the maintenance of telomere length and the presence of young memory T cells in the administered T cells [12]. However, preparation of the administered cells involves ex vivo expansion, leading to telomere shortening, loss of memory phenotype, and the induction of exhaustion [13]. To address these challenges, we developed induced pluripotent stem cells from antigen**-**specific T cells (T**-**iPSCs) and then differentiated them into T cells (iPS**-**T cells), which show less exhaustion phenotype [14]. This innovative approach holds promise in overcoming the limitations associated with ex vivo amplification and provides a potential solution for enhancing the effectiveness of immunotherapeutic interventions [14, 15].

## Utilization of iPSCs as a platform for immunotherapy

iPSCs, like embryonic stem cells (ESCs), possess unique stem cell characteristics, including pluripotency and ex vivo self**-**renewal, which enable their differentiation into various organs and cell types. However, unlike ESCs, which are derived from the inner cell mass of blastocysts, iPSCs do not have ethical concerns regarding embryo destruction, as they can be generated from easily accessible cells such as skin or blood. iPSCs were first created by introducing the Yamanaka factors (OCT3/4, Klf4, Sox2, and c**-**Myc) into somatic cells, which reprogrammed the cells to an undifferentiated state from which they can be differentiated into diverse cell types [16]. This versatility has led to widespread application in various research fields. Another advantage of iPSCs is their facile genetic manipulation. Various genome editing techniques, such as TALEN, CRISPR/Cas9, and viral vectors introduce genes into iPSCs, enabling gene knock**-**in and knock**-**out [17–19]. This capability not only enhances cellular functions in regenerative medicine but also aids in exploring drug targets for modeling and treating pathologies resulting from genetic mutations. The potential of iPSCs in both basic and applied research on various diseases is highly anticipated.

In recent years, immunotherapy has emerged as a promising cancer treatment strategy, alongside surgery, chemotherapy, and radiation therapy. However, one of the challenges in current immunotherapy is the preparation and amplification of patient**-**derived peripheral blood cells, which heavily depend on the manufacturing process and frequently delay treatment [20]. One reason for this challenge is the use of patient**-**derived peripheral blood cells to prevent immune rejection. This rejection occurs due to differences in HLA types between the recipient and the donor, where the host cells recognize, and attack transplanted cells as foreign bodies [21]. Achieving a complete match in HLA type is extremely rare, occurring only once in several hundred to several thousand individuals, making allogeneic transplantation with HLA heterozygous cells a challenge. In contrast, the use of HLA**-**homozygous cells, particularly those derived from iPSCs, has gained momentum in immunotherapy, because matching only one allele of the recipients’ HLAs is sufficient, making it applicable to a broader population [22]. Specifically, the establishment and storage of immune cells derived from HLA**-**homozygous iPSC lines could address the current challenges listed above. The use of iPSCs as an “off**-**the**-**shelf” cell source, readily available for immediate supply to patients regardless of their HLA type, is expected to advance regenerative immunotherapy. Therefore, we established iPS**-**T cells from antigen**-**specific T**-**iPSCs.

We successfully generated T**-**iPSCs and differentiated them into iPS**-**T cells through staged co**-**culture with feeder cells, namely C3H10T1/2 and OP9/DLL1, under appropriate cytokine conditions [14]. Antigen**-**specific T cells undergo irreversible gene recombination during their differentiation stages. While the reprogramming to T**-**iPSCs resets the epigenomic information of the cells, the genomic information is essentially retained. Therefore, genomic information of TCRs, which determine antigen specificity by recombination, is retained in T**-**iPSCs. This result suggests that iPS**-**T cells do not undergo TCR reconstitution and retain the same antigen specificity as the original T cells. In addition, iPS**-**T cells exhibit elongated telomeres compared to the original T cells, revealing enhanced antigen**-**specific cytokine production and proliferative capacity. Therefore, iPS**-**T cells leverage the stem cell properties of iPSCs to provide a stable supply of functional T cells, thereby presenting a novel platform for immunotherapy.

## Establishment of an efficient differentiation method for functional CD8αβ + iPS-T cells

In recent years, several research groups, including ours, have reported the induction of T**-**cell lineage cells from iPSCs in vitro [14, 23–27]. The differentiation of iPSCs involves the intermediate state of hematopoietic progenitor cells (HPCs). Although CD8 molecules are primarily composed of heterodimers (CD8αβ) [24, 28], iPS**-**T cells mostly exhibit homodimers of CD8αα. CD8αβ + T cells have been reported to enhance the binding affinity to TCR and major histocompatibility complex (MHC)**-**peptide complexes compared to CD8αα + T cells [29], leading to improved cytotoxic activity. Consequently, to produce CD8αβ + T cells with high reactivity to antigens, we developed a new method to produce CD8αβ + iPS**-**T cells (iCD8αβ + T cells).

T cells undergo differentiation and maturation from hematopoietic stem cells in the bone marrow and thymus in vivo. T**-**cell maturation requires education through MHC selection at the stage of double**-**positive (DP) cells expressing CD4 and CD8 in the thymus cortex [30, 31]. Positive selection involves the removal of cells unable to recognize self**-**MHC**-**peptide complexes in the thymic cortex, and negative selection induces apoptosis in cells that strongly bind to self**-**antigens in the thymic cortex**-**medulla region, thus promoting T cell maturation. This means that T cells are judged to possess functional TCRs without damaging self**-**tissues before differentiating into CD8αβ + T cells [31]. To mimic the thymic environment, we established a culture system under conditions simulating the thymus and enhanced the binding affinity to HLA**-**peptide complexes in CD8αβ + T cells by providing a TCR stimulation. Glucocorticoids, particularly dexamethasone (DEX), have been implicated in strengthening TCR signaling in vivo [32, 33]. Therefore, we attempted to establish an efficient differentiation method (DEX method) during the TCR stimulation. Indeed, when differentiating T**-**iPSCs in the presence of DEX, we efficiently induced iCD8αβ + T cells without inhibiting differentiation and observed an increase in IL**-**7R expression, which is associated with lymphocyte proliferation and survival [34]. Surprisingly, DEX, typically an immunosuppressive agent, not only enhanced IL**-**7R expression in DP cells but also supported the differentiation of iCD8αβ + T cells with high reactivity to antigens. However, a gene analysis revealed that iCD8αβ + T cells induced by DEX exhibited a loss of antigen**-**specific TCR expression, suggesting that DEX induced a gene recombination of TCR genes. This result suggests that the DEX method produced iCD8αβ + T cells with high antigen**-**binding affinity; however, it also led to a reduction in antigen specificity, as seen by the genetic recombination of TCRs.

Previous studies have shown that RAG2 gene knockout eliminates T**-**cell maturation and TCR gene recombination in mice [35]. Therefore, we used the CRISPR/Cas9 system to knock out the RAG2 gene at the iPSC stage and investigated antigen specificity following differentiation with the DEX method [34]. We found that RAG2 knockout T**-**iPSC lines maintained the ability to differentiate into iCD8αβ + T cells without inducing TCR recombination even in the DEX condition. RAG2**-**knockout iCD8αβ + T cells also showed high antigen**-**binding affinity and cytotoxicity in vitro. Further, they suppressed tumor progression and prolonged animal survival in vivo. To apply iPSCs (not just T**-**iPSCs) to immunotherapy, we also produced antigen**-**specific iCD8αβ + T cells from HLA**-**homozygous iPSC stocks by expressing TCR recognizing WT1 (Wilms’ tumor gene 1) antigen, which is expressed in various cancer types [36], to the iPSCs. These findings indicate that RAG2 knockout in the DEX method enables the generation of functional iCD8αβ + T cells with enhanced antigen specificity both in vitro and in vivo [34].

## Establishment of feeder-free differentiation

As stated above, one of the advantages of using iPSCs in immunotherapy is the ability to produce and store the T cells prior to demand. However, conventional differentiation methods are not efficient or appropriate for the large**-**scale production of T cells. Moreover, the use of feeder cells in the differentiation process and the requirement for GMP**-**grade cells without viral contamination for clinical applications have led to high costs or, in some cases, impractical implementation of the therapy. Therefore, our laboratory has focused on developing a method to produce a large number of T cells without the need for feeder cells [37].

To establish a feeder**-**free differentiation method for T cells, we divided the differentiation of iPSCs to T cells into four stages: iPSCs, HPCs, DP cells, and iCD8αβ + T cells. We explored proteins and compounds to replace feeder cells and successfully developed a feeder**-**free differentiation method. Additionally, adding factors crucial for thymic function, such as SDF1α, which induces early T cell development through CXCR4 [38], and a p38 inhibitor, which promotes cell proliferation by concentrating functional subsets of T cells [39], improved the efficiency of the T cell differentiation. To evaluate the functionality of the T cells produced using this method, we expressed a TCR recognizing WT1 antigen in iPSCs and differentiated the cells to iCD8αβ + T cells. These iCD8αβ + T cells exhibited antitumor efficacy in vivo, suppressing tumor volume growth and extending the survival period of the treated mouse group. Using T**-**iPSCs or TCR**-**expressing iPSCs as starting materials improved the efficiency of T cell differentiation under feeder**-**free conditions. This differentiation culture method may offer safer iPS**-**T cells without animal**-**derived components and paves the way for the mass production of iCD8αβ + T cells.

## Functional enhancement through genetic modification of iCD8αβ + T cells

**Fig. 1:**
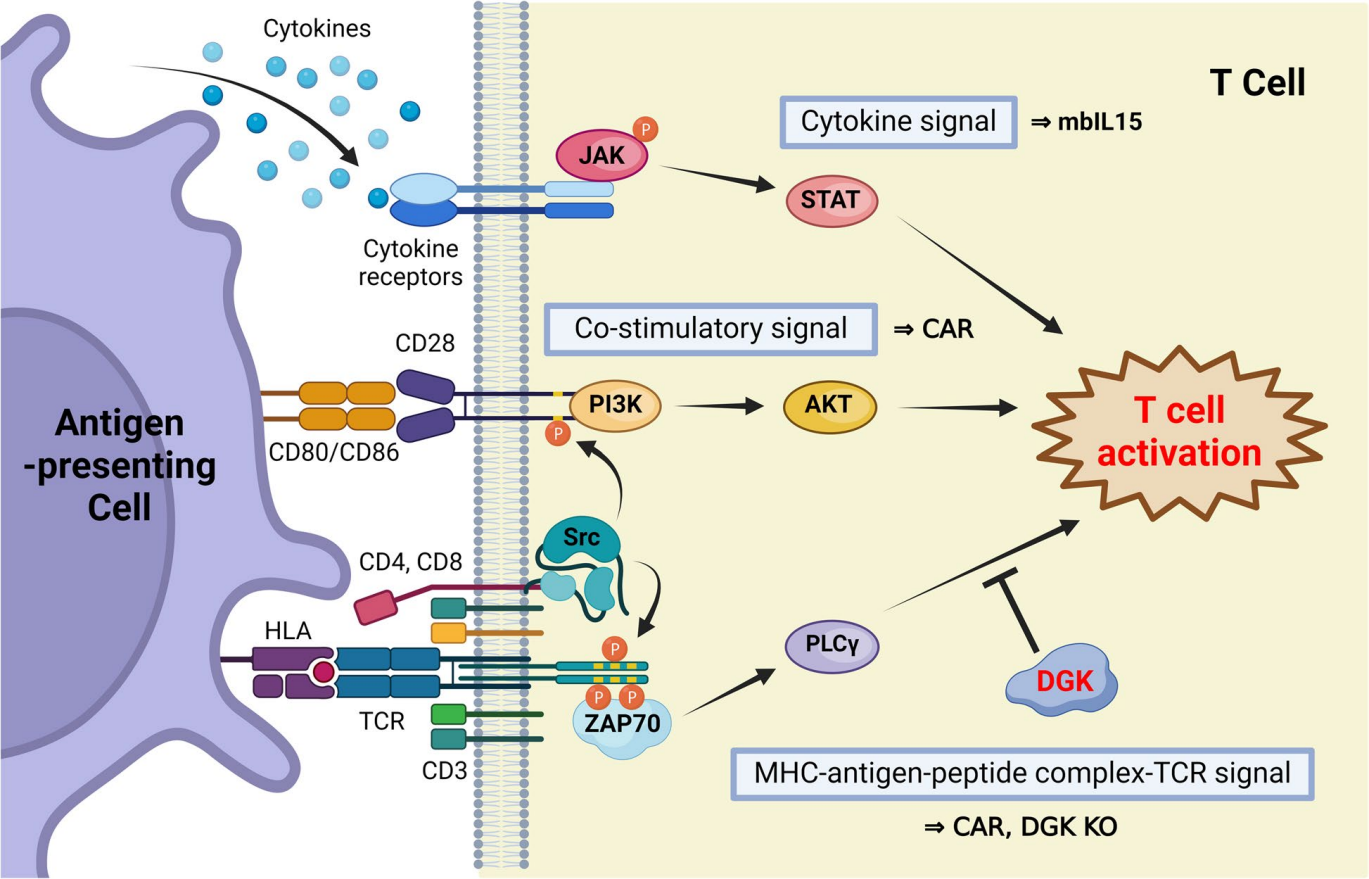
Mechanisms and improvement strategies for T cell activation regulation. T cell activation is primarily controlled by three signals: MHC**-**antigen**-**peptide complex**-**TCR signaling, co**-**stimulatory signaling, and cytokine signaling. Each signal initiates signaling cascades through phosphorylation events, such as ITAM (intracellular region: yellow area) phosphorylation. T cell activation promotes differentiation and proliferation and enhances effector functions. CARs possess a structural combination of TCR and co**-**stimulatory molecules. Strategies considered to enhance each signal include DGK double knockout (KO) and the introduction of mbIL15

The activation of T cells is primarily regulated by three signals [40, 41] (Fig. 1). The first signal is stimulation through the binding of HLA**-**peptide complexes with TCR. TCR forms a complex with CD3 on the cell membrane, and upon antigen binding, signaling cascades are initiated, starting with the phosphorylation of amino acid sequences called ITAM in the CD3 intracellular domain. Subsequently, the activation of tyrosine kinases, such as ZAP70, enhances signal responses, ultimately leading to T cell activation. The second signal is provided by co**-**stimulatory signals such as CD28. Co**-**stimulatory molecules play a crucial role in determining T cell activation or inactivation, with CD28 being an activating co**-**stimulatory molecule that transmits signals necessary for the binding of B7 molecules (CD80/CD86) on antigen**-**presenting cells. The third signal involves stimulation through cytokine signaling. Cytokine receptors that bind to cytokines, such as IL**-**2, activate the JAK/STAT pathways and promote cell proliferation. These three stimuli are essential for T**-**cell activation. CARs are artificially engineered receptors that combine signals 1 and 2 at the antigen recognition site (scFc), co**-**stimulatory molecules (CD28, 4**-**1BB, etc.), and the intracellular domain (CD3ζ). CAR**-**based immunotherapy has shown significant therapeutic efficacy against hematologic malignancies, and there is growing anticipation for its combination with iPS**-**T cells [11, 42–44]. In response, we generated anti**-**CD19 CAR**-**expressing iCD8αβ + T cells (iCAR**-**T cells) [45]. To induce the third critical factor for T cell activation, the cytokine signal, we expressed the membrane**-**bound IL**-**15/IL**-**15Rα (mbIL15) gene [46] in iCAR**-**T cells with a retroviral vector and evaluated their function in vitro and in vivo [37]. iCAR**-**T cells demonstrated CD19**-**dependent cytotoxic activity, and in vivo experiments revealed longer survival compared to the control group, demonstrating comparable therapeutic effects as primary T cells expressing CARs (pCAR**-**T cells).

Subsequently, we targeted solid tumors with poor therapeutic responses and genetically enhanced iCAR**-**T cells by editing crucial genes related to T cell activation signals. A detailed analysis of the iCAR**-**T cell function revealed a deficiency in the phosphorylation of ERK, a downstream molecule in the CAR signaling pathway when compared to pCAR**-**T cells [45]. To enhance the effector function of iCAR**-**T cells, we strengthened the first T cell activation signal, TCR stimulation, by modulating diacylglycerol kinase α (DGKα) and DGKζ, two proteins known to inhibit TCR signals. By knocking out DGKα and DGKζ using the CRISPR/Cas9 system, we created iCAR**-**T cells with enhanced TCR signals, which were evaluated in a subcutaneous xenograft mouse model. The results demonstrated improved tumor growth control and survival in the group treated with modified iCAR**-**T cells compared to conventional iCAR**-**T cell treatment or unmodified pCAR**-**T cell treatment.

Finally, we evaluated iCAR**-**T cells gene modification with DGKα and DGKζ double knockout and expression of mbIL15 [45]. These iCAR**-**T cells showed improved antitumor efficacy against solid tumors, such as ovarian cancer and hepatocellular carcinoma, which is challenging for cancer immunotherapy compared to single gene**-**modified iCAR**-**T cells. This gene combination improved mice survival suggesting that optimization of the gene editing of iCAR**-**T cells could enhance further antitumor efficacy against solid tumors.

## Immunogenicity reduction of iCD8αβ + T cells for clinical application

We have been enhancing the therapeutic efficacy of iCAR**-**T cells by improving the T cell activation pathway and promoting signal amplification through the inhibition of immune checkpoint molecules. However, challenges persist in the widespread adoption of immunotherapy using iPSCs. One major challenge is immune rejection due to HLA incompatibility [47]. To address this issue, the Center for Cell Research and Application, Kyoto University, initiated the “iPS Cell Stock Project” in 2013, with the aim of accumulating iPSCs derived from HLA**-**homozygous donors [22]. By advancing this project and stocking multiple HLA-homozygous iPSC lines, it is possible to rapidly provide cells to individual patients in terms of HLA type, differentiation potential, gender, and other concerns. This project is designed to promote “off**-**the**-**shelf” cellular therapies. However, establishing HLA**-**homozygous iPSC lines that satisfy the worldwide population is a challenging task. For example, to cover 90% of the Japanese population, it is estimated that at least 150,000 donors must be screened [48]. Furthermore, the frequency of HLA allelic variations varies among races and ethnicities, making the establishment of HLA**-**homozygous iPSC lines that cover people globally a challenging and time**-**consuming task [49]. In response, we are preparing low**-**immunogenic iPSCs that are less susceptible to attacks from the recipient’s immune cells using genome**-**editing technology. The transplantation of allogeneic T cells inherently carries the risk of eliciting immune responses from the host, primarily by CD8 + T cells recognizing HLA class I molecules, CD4 + T cells recognizing HLA class II molecules, and NK cells sensing the absence of any HLA expression. While these recognition mechanisms contribute to the removal of foreign entities, such as bacteria and cancer, they can be detrimental during allogeneic cell transplantations because of possible immune rejection.

Considering this context, we reported two methods for creating iPSC lines with a reduced risk of rejection using genome**-**editing technology [50]. One method involves the removal of only HLA**-**A, HLA**-**B, and HLA**-**C from one allele of HLA heterozygous iPSCs (HLA pseudo**-**homozygous iPSCs). iPSCs produced by this method demonstrate reduced susceptibility to T cell attacks. Additionally, to cover a wide range of people with a small iPSC stock, we successfully created low**-**immunogenic iPSCs by editing genes to knockout HLA**-**A and HLA**-**B but not HLA**-**C. These low**-**immunogenic iPSCs were found to evade attacks not only from T cells with HLA mismatch but also from NK cells. Furthermore, using a mouse model transplanted with CD8 + T cells, we confirmed that the survival rate of these cells was higher than that of non**-**edited cells, indicating a reduced vulnerability to attacks by CD8 + T cells. By retaining HLA**-**C, we were able to suppress immune rejection reactions. HLA**-**C retention is a potential solution to achieving wide**-**scale iPSC**-**based immunotherapy.

**Fig. 2:**
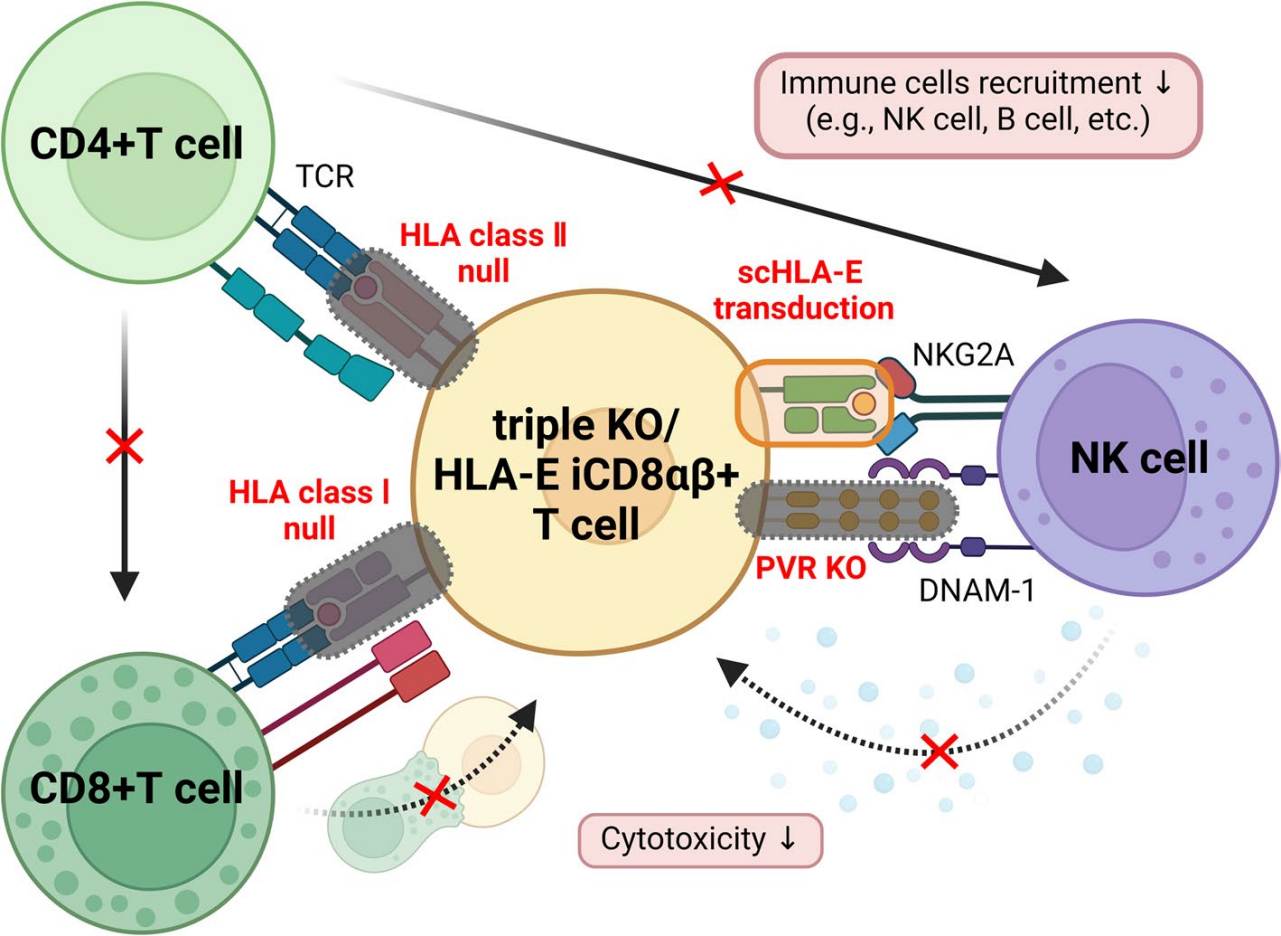
Overview of reduced immunogenicity in iCD8 T cells through gene editing. To circumvent immune rejection, genetic editing is employed to knock out HLA class I and HLA class II molecules, rendering iCD8αβ + T cells unrecognizable by T cells through TCR**-**mediated recognition. Immune responses from NK cells are addressed by introducing the HLA**-**E gene and knocking out NK cell activation ligands expressed on the surface of iCD8αβ + T cells (triple KO). These gene editing strategies are effective at inhibiting the direct recognition of host immune cells and the mobilization of immune cells via CD4 + T cells, thus suppressing cytotoxic activity and preventing the elimination of tKO/E**-**iCD8αβ + T cells

However, the complete suppression of NK cell activity was not achieved using this method. Therefore, we considered additional genetic modifications, including the knockout of HLA class I and II molecules and the modification of ligands involved in NK cell activation (Fig. 2) [51]. We initially embarked on the generation of iPSCs capable of evading reactions from T**-**cell populations through the elimination of HLA class I and class II molecules. This approach involved the creation of iPS cells, denoted as double knockout iPSCs (dKO**-**iPSCs), in which the proteins B2M and C II TA were knocked out using the CRISPR/Cas9 system. The differentiated iCD8αβ + T cells (dKO**-**iCD8αβ + T cells), when co**-**cultured with HLA**-**homologous T cells, show less reactivity to recipient T cells than iCD8αβ + T cells. Next, we focused on HLA**-**E and PVR, a regulatory molecule in NK cells known to control the cytotoxic activity of some NK cells [52]. We introduced single**-**chain HLA**-**E (scHLA**-**E) using lentivirus and PVR gene knockout using the CRISPR/Cas9 system on dKO**-**iPSCs and differentiated them to iCD8αβ + T cells (tKO/E**-**iCD8αβ + T cells). tKO/E**-**iCD8αβ + T cells showed better survival in co**-**culture with NK cells than iCD8αβ + T cells in vitro. Finally, to ensure that tKO/E**-**iCD8αβ + T cells could effectively evade immune rejection from the recipient and maintain their cytotoxicity toward target cells in vivo, anti**-**CD20 CAR was introduced. These cells were intravenously injected into NSG mice with healthy donor**-**derived immune cells. The anti**-**CD20 CAR**-**expressing tKO/E**-**iCD8αβ + T cells showed therapeutic efficacy against CD20**-**expressing target cells and evaded the immune cells longer than iCD8αβ + T cells in vivo. This study demonstrates that tKO/E**-**iCD8αβ + T cells have lower immunogenicity than HLA pseudo-homozygous or HLA**-**C**-**retained iCD8αβ + T cells; therefore, tKO/E**-**iCD8αβ + T cells are anticipated to advance T cell therapies.

## Conclusions

In this review, we have discussed the characteristics and research findings of iPSCs for future T cell therapies. Due to the pluripotency and ease of genetic editing iPSCs, there is an anticipation for the development of T cells with enhanced functionalities. While our discussion has primarily focused on T cells, other immune cells using similar strategies are expected. Our group has contributed to this aspect of immunotherapy too [23, 26, 53–55]. Notably, CAR**-**expressing NK cells have demonstrated utility in non**-**clinical trials, with their effectiveness recognized since 2020 in phase I clinical trials [56].

Moving forward, overcoming the clinical hurdles of immunotherapy using iPSCs is expected to be facilitated by the development of low immunogenicity iPSCs. Low**-**immunogenicity iPSCs are poised to address manufacturing challenges and promote the widespread adoption of iPSC**-**based immunotherapy. The progress in research on a diverse array of regenerated immune cells not only contributes to the establishment of therapeutic approaches using individual immune cells but also holds the potential for synergistic effects through combination therapies, which may lead to treatments for other diseases beyond cancer. However, it is crucial to overcome the severe side effects observed in previous T cell therapies, such as graft**-**versus**-**host disease and cytokine release syndrome (CRS). We earnestly hope to achieve the realization of iPSC**-**derived immune cell therapy and overcome these outstanding issues in the near future.

## Data Availability

Not applicable.
